# DDB1 Regulates Sertoli Cell Proliferation and Testis Cord Remodeling by TGFβ Pathway

**DOI:** 10.3390/genes10120974

**Published:** 2019-11-26

**Authors:** Wei Zheng, Jabeen Nazish, Fazal Wahab, Ranjha Khan, Xiaohua Jiang, Qinghua Shi

**Affiliations:** 1 School of Life Sciences, Reproductive and Genetic Branch, Division of Life Sciences and Medicine, CAS Center for Excellence in Molecular Cell Science, University of Science and Technology of China, Hefei 230027, Anhui, China; ustczw@163.com (W.Z.); jabeen@mail.ustc.edu.cn (J.N.); fwahab@ustc.edu.cn (F.W.); rhanjha@mail.ustc.edu.cn (R.K.); 2 Division of Molecular Medicine, Hefei National Laboratory for Physical Sciences at the Microscale, University of Science and Technology of China, Hefei 230026, Anhui, China

**Keywords:** *Ddb1*, Sertoli cell, testis cord, spermatogenesis, TGFβ

## Abstract

Testis cords are the embryonic precursors of the seminiferous tubules. Development of testis cords is a key event during embryonic testicular morphogenesis and is regulated by multiple signaling molecules produced by Sertoli cells. However, the exact nature and the cascade of molecular events underlying testis cord development remain to be uncovered. In the current study, we explored the role of DNA damage binding protein 1 (DDB1) in Sertoli cells during mouse testis cord development. The genetic ablation of *Ddb1* specifically in Sertoli cells resulted in the compromised Sertoli cell proliferation and disruption of testis cord remodeling in neonatal mice. This testicular dysgenesis persisted through adulthood, resulting in smaller testis and low sperm production. Mechanistically, we observed that the DDB1 degradation can stabilize SET domain-containing lysine methyltransferase 8 (SET8), which subsequently decreases the phosphorylation of SMAD2, an essential intracellular component of transforming growth factor beta (TGFβ) signaling. Taken together, our results suggest an essential role of *Ddb1* in Sertoli cell proliferation and normal remodeling of testis cords via TGFβ pathway. To our knowledge, this is the first upstream regulators of TGFβ pathway in Sertoli cells, and therefore it furthers our understanding of testis cord development.

## 1. Introduction

Seminiferous tubules are the basic structural and functional components of the adult mammalian testis. During embryonic development, seminiferous tubules arise from the testis cords [1,2]. Morphological analysis in mouse embryo reveals that testis cords are formed as a consequence of complex series of parallel transverse loops, separated from each other by interstitial cells at about 14.5 dpc (days post coitum) [1,2]. Subsequently, through the elongation and expansion of the initial testis cords, a significant number of small cross-sections of the cord structure are formed at about 0 dpp (days post-partum) [3,4,5]. Of important note, any delay or disruption of testis cord development results in the gonadal dysgenesis [1,6]. Nevertheless, the molecular factors and signaling pathways involved in the development of testis cord are still poorly characterized.

Sertoli cells, the major somatic cells of testis, consist the main epithelium and sequester germ cells within the lumen of testis cords [5]. The expansion of perinatal Sertoli cell population is essential to testis morphogenesis by guiding testis cord development [1,3,5,7]. Increasing evidences demonstrate that transforming growth factor beta (TGFβ) superfamily plays a vital role in regulation of the Sertoli cell proliferation [8,9,10,11]. For example, specific deletion of *Smad4*, a central component of TGFβ signaling, in Sertoli cells, led to the Sertoli cell proliferative defects and testis cord dysgenesis [9]. However, to date, the upstream regulators of TGFβ pathway in Sertoli cells have not been fully elucidated.

DDB1 (DNA damage-binding protein 1) is an important adapter protein for CRL4 (Cullin-RING finger ligase 4) ubiquitin ligase during DNA damage repair, cell cycle, and transcription processes [12,13,14,15,16]. Recent studies revealed that in genetic mouse models, the deletion of *Ddb1* in germ cells leads to oocyte loss in female and spermatogonial stem cell (SSC) deficiency in male [17,18]. However, to date, the role of *Ddb1* in Sertoli cells, especially for testis cord development has not been reported.

In this study, in order to explore the role of *Ddb1* in fetal Sertoli cells, we deleted *Ddb1* specifically in Sertoli cells by *AMH*-Cre. The Sertoli cell proliferation was decreased because of the compromised TGFβ pathway. Remarkably, the *Ddb1* deficiency in fetal Sertoli cells resulted in disruption of testis cord remodeling and, finally, small testis in adult.

## 2. Materials and Methods 

### 2.1. Experimental Mice

*Ddb1*^fl/fl^ mice, homozygous for floxed allele of *Ddb1*, and *AMH*-Cre transgenic mice were described previously [19,20]. These mouse strains were maintained on a mixed C57BL/6J/129 genetic background. All mice were kept under controlled photoperiod conditions (lights on 08:00–20:00) and supplied with food and ddH_2_O. All animal studies were conducted in accordance with the guidelines approved by Institutional Animal Care Committee of University of Science and Technology of China (USTCACUC1301021).

### 2.2. Sertoli Cell Isolation

Sertoli and germ cells from 14 dpp mice were isolated, as we described previously [21,22]. Briefly, testes were decapsulated and 4 to 6 mice were pooled together, then seminiferous tubules washed with phosphate-buffered saline (PBS) and incubated with 2 mg/mL collagenase IV (Sigma, C1889, St. Louis, MO, USA) in DMEM/F12 (HyClone, SH40007-13, Logan, UT, USA) for 10 min at 37 °C, and seminiferous tubules were further digested with 0.05% trypsin (Sigma, T8003, St. Louis, MO, USA) and 1 mg/mL DNase I (Sigma, D4527, St. Louis, MO, USA) for 30 min at 37 °C. The final dispersed cells were placed into culture dishes in DMEM/F12 containing 10% fetal bovine serum (HyClone, SV30087-02, Logan, UT, USA) and incubated at 37 °C with 5% CO_2_. After 24 h culture, the suspension cultured cells were collected, and the attached cells were treated with a hypotonic solution (20 mM Tris, pH 7.4) for 3 min and harvested for Western blotting. On the basis of the cell attachment differences, the attached cells were mainly Sertoli cells, and the suspension cultured cells were mainly germ cells.

### 2.3. Western Blotting

Western blotting was carried out, as described previously [22] and primary antibodies against DDB1 (1:300; Bethyl, A300-462A, Montgomery, TX, USA), glyceraldehyde-3-phosphate dehydrogenase (GAPDH; 1:1000; Millipore, MAB374, Burlington, MA, USA), SET8 (1:200, Abcam, ab3798, Cambridge, UK), SMAD2 (1:100; Santa Cruz; sc-8332, Dallas, TX, USA) and p-SMAD2 (1:300, Cell Signaling Technology, 3108P, Danvers, MA, USA) were used. The bands were quantified using the Gel-Pro Analyzer 4.0 computer program.

### 2.4. Histological Analysis

The average diameter of the seminiferous tubules was determined by measuring the diameter of 50 tubular cross-sections by means of image analysis [23]. For stereological analysis, testes were embedded, cut into sections (20 mm) and stained with hematoxylin. The total testis volume was estimated using the Cavalieri principle [24]. The number of Sertoli cells and germ cells in each testis was counted by the optical dissector technique [25]. Gonocytes were recognized by their large round nuclei and clearly identifiable cellular surroundings, and Sertoli cells were recognized by their irregularly shaped nuclei and hardly detectable cellular surroundings [23,26,27].

The epididymis and vas deferens were removed from control and *Ddb1* cKO mice at 10 weeks of age, incised several times, and incubated in 1 mL buffer containing 75 mM NaCl, 24 mM EDTA, and 0.4% bovine serum albumin (Sigma, A2058, St. Louis, MO, USA) at 37 °C for 30 min to allow sperm release. Sperm were collected after a nylon-mesh filtration and counted with a hemocytometer.

### 2.5. BrdU Labeling

A solution of 5 mg/mL Bromodeoxyuridine (BrdU, Sigma, B9285, St. Louis, MO, USA) was prepared in sterile saline. Then, 18 dpc pregnant female mice and 0 dpp newborn mice were injected with BrdU (50 mg/kg) and sacrificed for further analysis 3 h after the injection.

### 2.6. Hematoxylin and Eosin (H&E) Staining and Immunostaining

The control and *Ddb1* cKO mice were euthanized by cervical dislocation. Testes were immediately fixed in Bouin’s solution for H&E staining or in 4% paraformaldehyde in PBS for immunohistochemistry/immunofluorescence. For the BrdU staining, before the antigen recovery, BrdU epitope was exposed by incubating the slides in 2N hydrochloric acid for 20 min at 37 °C, then, neutralize by incubating in borate buffer (0.1 M) for 15 min at room temperature. Subsequently, the standard staining procedure was carried out, as described previously [22]. Primary antibodies for DDB1 (1:100; Bethyl, A300-462A, Montgomery, TX, USA), DDX4 (1:200; Abcam, ab13840, Cambridge, UK), SOX9 (1:200; Millipore, AB5535, Burlington, MA, USA), and BrdU (1:100; Thermo, MS-1058-P0, Waltham, MA, USA) were used for immunostaining. Next, horseradish peroxidase (HRP) conjugated Donkey anti-Rabbit IgG (1:200; Abcam, ab6802, Cambridge, UK) was used for immunohistochemistry, or Alexa Fluor 488-conjugated donkey anti-mouse (1:250; Molecular Probes, A21121, Eugene, OR, USA) and 555-conjugated donkey anti-rabbit (1:250; Molecular Probes, A31572, Eugene, OR, USA) IgG antibodies were used for immunofluorescence. To reduce inter-experiment variations, testes from control and cKO mice were processed simultaneously. All images were captured using a Nikon Eclipse 80i microscope equipped with a digital camera (Nikon DS-Ri1 for H&E and immunohistochemistry or Hamamatsu C4742-80 (Hamamatsu, Japan) for immunofluorescence). 

### 2.7. Statistical Analysis

The mean diameter of testis cords, the mean number of tubules per transverse section/Sertoli cells or germ cells per testis, testis weight, sperm number, and Sertoli cell proliferation ratio were compared between control and cKO mice using Student’s t-test. Results are presented as mean ± S.E.M and *p* < 0.05 was considered as a statistical significance.

## 3. Results

### 3.1. DDB1 Expression and Localization in Testes

**Figure 1:**
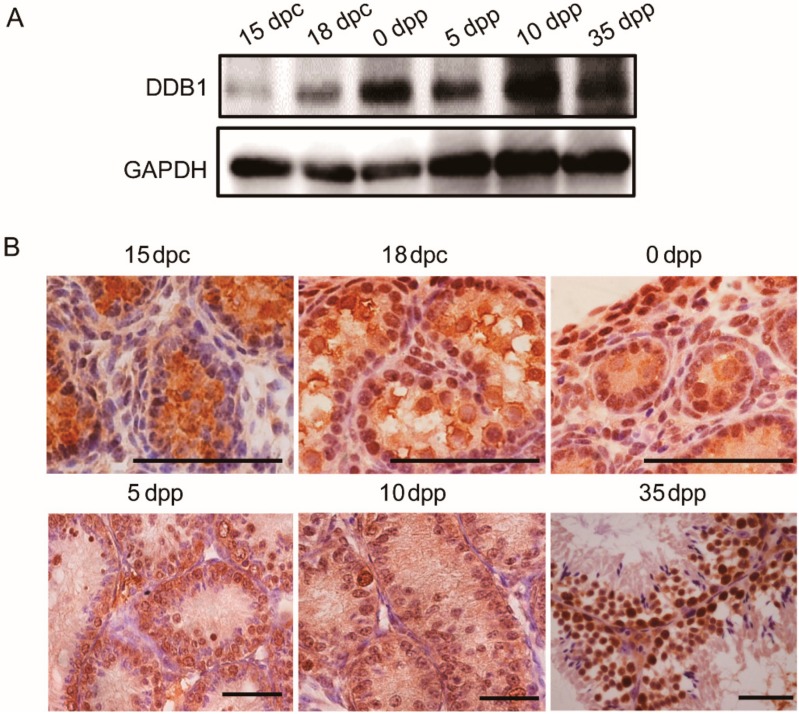
Expression and localization of DDB1 in pre- and postnatal mouse testes. (**A**) A representative image shows DDB1 protein levels in mouse testes. GAPDH was used as loading control; (**B**) Representative images show cellular localization of DDB1 in testes at indicated ages. Brown represents DDB1 and blue represents nuclei and scale bars = 50 μm.

To determine the expression profile of *Ddb1* during testis development, the DDB1 protein level was analyzed by Western blotting. We found that the level of DDB1 in testes was very low in 15 dpc but increased from 18 dpc and climbed strikingly in the newborn mice (0 dpp), then remained relatively constant until 35 dpp (Figure 1A). Furthermore, based on the immunohistochemistry staining, DDB1 was ubiquitously localized in the nuclei of perinatal and juvenile testes (Figure 1B). 

### 3.2. Genetic Deletion of Ddb1 in Sertoli Cells

**Figure 2:**
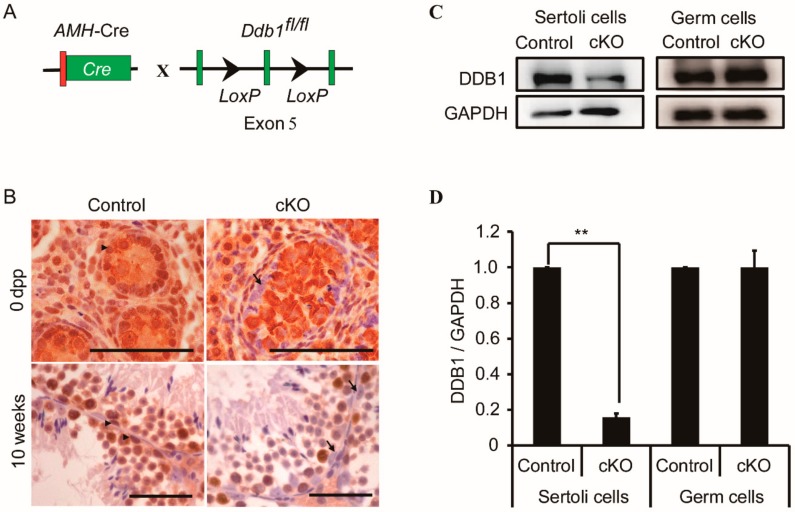
Specific deletion of *Ddb1* in Sertoli cells. (**A**) The schematic diagram showing the generation of *Ddb1* cKO mice; (**B**) immunostaining of DDB1 in control and cKO testicular sections, Arrowheads indicate DDB1 positive Sertoli cells and arrows indicate DDB1 negative Sertoli cells, scale bars = 50 μm; (**C**) Western blotting analysis of DDB1 in isolated Sertoli and germ cells from 14 dpp control and cKO mice, GAPDH was used as loading control; (**D**) quantitative results of Western blotting experiments as shown in (C). The DDB1 protein level was normalized against GAPDH. Control group data were set as 1. Data were presented as mean ± S.E.M. ** *p* < 0.01, Student’s t-test.

To explore the roles of *Ddb1* in Sertoli cells, we generated *Ddb1* conditional knockout (*Ddb1* cKO) mice in which *Ddb1* was specifically deleted in Sertoli cells by crossing *Ddb1*^fl/fl^ mice with the *AMH*-Cre mice (Figure 2A). Immunohistochemistry was performed to confirm the deletion efficiency of *Ddb1* in Sertoli cells and the results demonstrated that in contrast to the strong DDB1 staining in control Sertoli cells, no DDB1 staining was observed in *Ddb1* cKO Sertoli cells (Figure 2B). To be noted, DDB1 signals were still found in germ cells of newborn and 10 weeks *Ddb1* cKO mice (Figure 2B). Furthermore, DDB1 protein level was significantly decreased in the isolated cKO Sertoli cells as compared to that in control, in contrast, a comparable level of DDB1 was detected in the germ cells of control and *Ddb1* cKO mice (Figure 2C,D and Figure S1).

### 3.3. Disturbed Morphogenesis of Testis Cords in Neonatal Ddb1 cKO Mice

**Figure 3:**
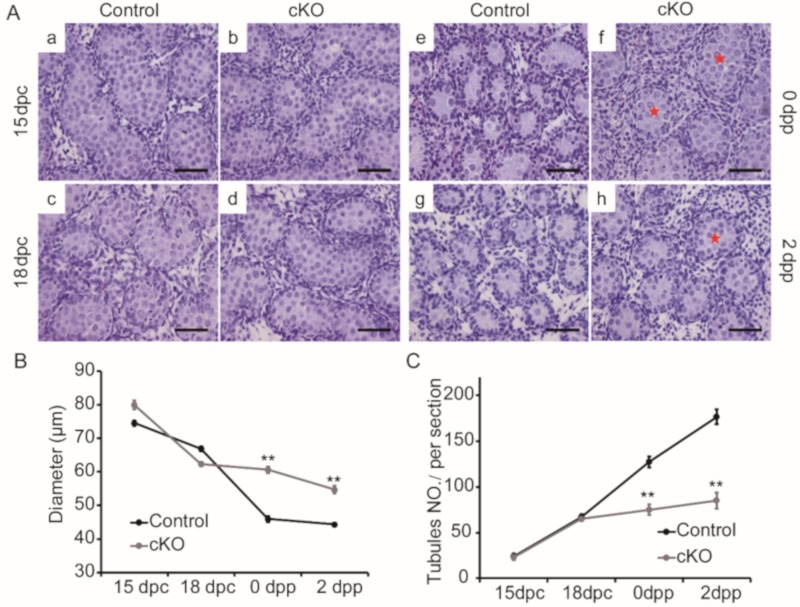
Disturbed morphogenesis of testis cords in neonatal *Ddb1* cKO mice. (**A**) Hematoxylin stained testicular sections from 15 dpc, 18 dpc, 0 dpp, and 2 dpp control and cKO mice. In 0 and 2 dpp cKO testes, the cords exhibit gross enlargement of cross-sectional diameter, red stars indicate much larger testis cords in cKO mice, scale bars = 50 μm; (**B**) the diameters of testis cords shown in (A); and (**C**) the number of testis cords per transverse section in (A). Data were presented as mean ± S.E.M. ** *p* < 0.01, Student’s t-test.

To determine the effects of *Ddb1* deletion in Sertoli cells, we firstly analyzed testis development at different time points. Based on the hematoxylin staining, no obvious differences in testis cord morphology were found between control and *Ddb1* cKO testes at 15 dpc and 18 dpc (Figure 3A(a–d)). However, compared to the controls, larger tubules were observed in 0 dpp and 2 dpp *Ddb1* cKO testes (Figure 3A(e–h)). Statistical analysis further confirmed larger testis cord diameter and a significant reduction of cord number in *Ddb1* cKO mice (Figure 3B,C). Interestingly, in 14 dpp and adult testes, the size of seminiferous tubules that are arisen from the testis cords, was comparable between the control and the *Ddb1* cKO mice (Figure S2C). Nevertheless, the testis size and sperm number in the epididymis decreased significantly and some vacuolated seminiferous tubules were observed in the *Ddb1* cKO mice (Figure S2).

### 3.4. Germ Cell Accumulation and Decreased Sertoli Cells in Neonatal Ddb1 cKO Mice

**Figure 4:**
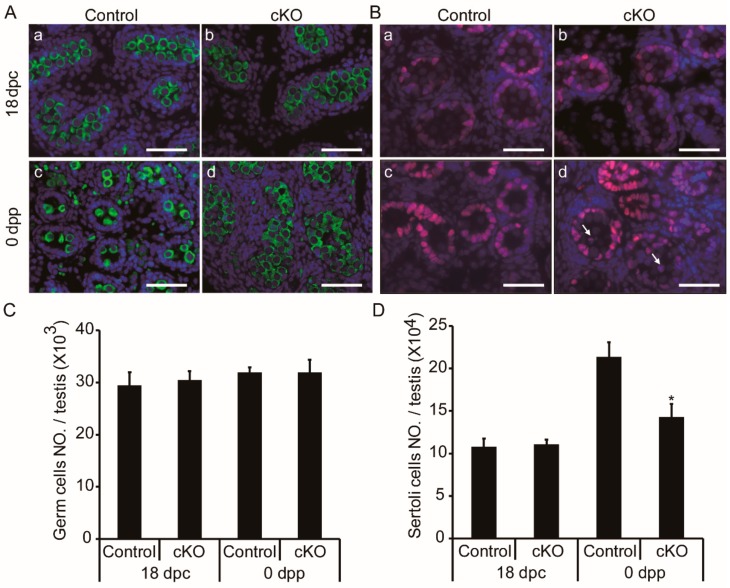
Accumulated tubular germ cells and decreased Sertoli cells in neonatal *Ddb1* cKO mice. (**A**) Testicular sections show germ cells (DDX4, green) in 18 dpc and 0 dpp mice, scale bars = 50 μm; (**B**) testicular sections show Sertoli cells (SOX9, red) in 18 dpc and 0 dpp mice, arrows indicate Sertoli cells that aberrantly located in the lumen of testis cords, scale bars = 50 μm; (**C**) the total number of germ cells per testis shown in (A); and (**D**) the total number of Sertoli cells in testicular section per testis as shown in (B). Data were presented as mean ± S.E.M. * *p* < 0.05, Student’s t-test.

To evaluate the cellular influences of *Ddb1* deletion on fetal testis cords, we performed immunofluorescence in 18 dpc and 0 dpp testes. On the basis of the DDX4 and SOX9 staining, no differences of germ and Sertoli cells were found between the control and the *Ddb1* cKO testes at 18 dpc, however, significant differences were observed in the 0 dpp *Ddb1* cKO mice (Figure 4A,B). Of particular note, there were less germ cells per tubule in the 0 dpp WT mice, whereas the overall germ cells in total testis had no obvious difference (Figure 4A(c,d),C). However, there was a decrease in the total Sertoli cell number in the *Ddb1* cKO mice as compared to the control (Figure 4D). Moreover, alteration in the localization of Sertoli cells was also observed in the *Ddb1* cKO testes. In the seminiferous tubules of control mice, Sertoli cells were located at or near the periphery, whereas in the *Ddb1* cKO testes some Sertoli cells were also seen in the lumen of the tubules (Figure 4B(c,d)). 

### 3.5. Sertoli Cell Proliferation Decreased in Neonatal Ddb1 cKO Mice

**Figure 5:**
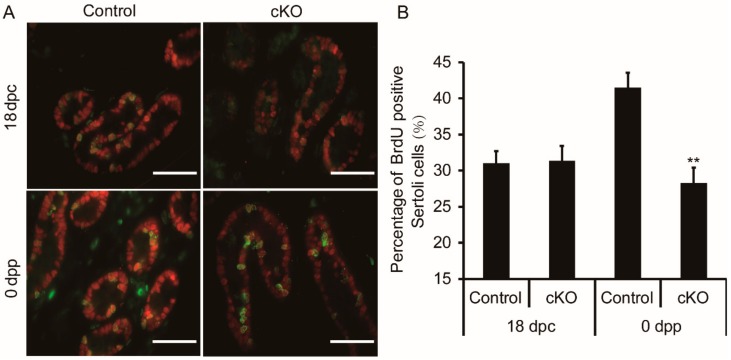
Decreased Sertoli cell proliferation in neonatal *Ddb1* cKO mice. (**A**) Immunofluorescence shows the proliferative (BrdU, green) and Sertoli cells (SOX9, red) in testes obtained from 18 dpc and 0 dpp mice, scale bars = 50 μm and (**B**) the percentage of BrdU-positive Sertoli cells shown in (A). Data were presented as mean ± S.E.M. ** *p* < 0.01, Student’s t-test.

To investigate the reason for Sertoli cell reduction in *Ddb1* cKO testes, BrdU incorporation was performed. On the basis of the BrdU and SOX9 double staining assay, we found that the percentage of BrdU positive Sertoli cells was similar in the control and cKO testes at 18 dpc, however, in 0 dpp, the percentage of double stained positive cells was decreased significantly in the *Ddb1* cKO testes as compared to the control testes (Figure 5).

### 3.6. Variable Expression of Proteins Associated with DDB1 in Ddb1 cKO Mice

**Figure 6:**
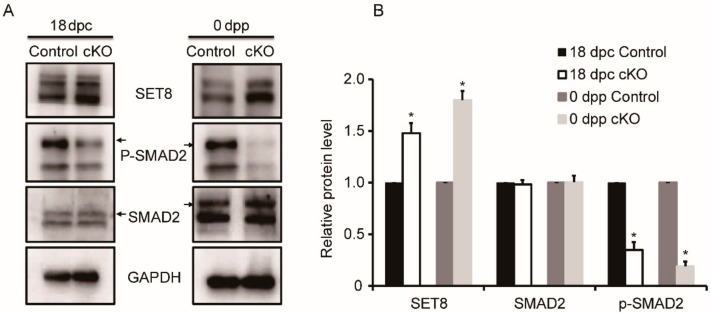
Variable expression of proteins associated with DDB1 in *Ddb1* cKO mice. (**A**) SET8, SMAD2, and p-SMAD2 expression in 18 dpc and 0 dpp mouse testes. GAPDH was used as loading control and (**B**) quantitative results of Western blotting shown in (A). The SET8, SMAD2, and p-SMAD2 protein level were normalized against GAPDH and were arbitrarily set as 1 in the control group. Data were presented as mean ± S.E.M. * *p* < 0.05, Student’s t-test.

As an adapter of ubiquitin ligase of CRL4, DDB1 mediates protein degradation through the SET8-TGFβ pathway during cell cycle regulation [28,29,30]. To investigate the involved pathways of *Ddb1* during testis cord development, the levels of SET8, SMAD2, and p-SMAD2 were investigated by Western blotting. Although no obvious change in SMAD2 expression was observed between the control and cKO testes, the level of p-SMAD2 was significantly decreased in the *Ddb1* cKO testes as compared to that in the control at 18 dpc and 0 dpp, additionally, the expression of SET8 was dramatically increased in both the 18 dpc and the 0 dpp *Ddb1* cKO testes (Figure 6A,B).

## 4. Discussion

In this study, we explored the role of DDB1 in Sertoli cells and found that *Ddb1* deficiency in Sertoli cells leads to compromised Sertoli cell proliferation and abnormal development of testis cords.

DDB1 is ubiquitously expressed in almost all testicular cell types and previous studies have revealed that it is essential for germ cell development [17,18]. To define the role of *Ddb1* in Sertoli cells, we generated Sertoli cell *Ddb1* deficient mice by crossing *AMH*-Cre transgenic mice with *Ddb1 floxp* mice (Figure 2A). To be noted, as the *AMH*-Cre transgene is expressed from 14.5 dpc onwards [20], excision of *Ddb1* occurs before the onset of testis cord coiling [3,4,5]. Consistently, the Sertoli cell expression pattern of DDB1 was abolished in the cKO testes based on immunohistochemical analysis. Additionally, we observed that the *Ddb1* deletion using *AMH*-Cre resulted in a drastic reduction of DDB1 protein level in the isolated Sertoli cells of cKO mice (Figure 2C), further confirming efficient deletion of *Ddb1* in these cells (Figure 2D).

Perinatal testis cords undergo a process of elongation and convolution. Intriguingly, a sharp increase of testicular DDB1 level is observed from 18 dpc to 0 dpp (Figure 1A). This coincidence of DDB1 protein expression pattern with the coiling of testis cord strongly suggests that it could play a role for the testis cord morphogenesis. Based on our observation, until 18 dpc, there is no obvious differences of testis morphology between the control and *Ddb1* cKO mice, indicating that DDB1 has no obvious effect on the initial testis cord formation (Figure 3). However, the neonatal *Ddb1* cKO mice still exhibit lots of simple transverse loop structure (Figure 3A(f)), reflecting severe testis cord elongation and convolution defects when *Ddb1* was deleted in Sertoli cells. Taken together, these findings suggest that DDB1 plays a vital role in the testis cord remodeling.

The dramatic morphological change of testis cords is presumably driven by the rapid proliferation of the Sertoli cells. Sufficient quantity of Sertoli cells are required to separate and surround the germ cells within the lumen [1,3,7,8]. In control neonatal mice, the Sertoli cell number per testis is much more than that in the *Ddb1* cKO mice and extensive testis cord elongation and coiling occurred. In contrast, as compared to the controls, the Sertoli proliferation rate in the 0 dpp *Ddb1* cKO mice is decreased by 40% (Figure 5). Therefore, we suggest that the significant reduction of Sertoli cell proliferation accounts for the stunted coiling of testis cords in cKO mice. Previously, it has been documented that the ratio of Sertoli cells to germ cells is relatively constant in mice [31]. Therefore, due to the reduction of Sertoli cells in the *Ddb1* cKO mice, the existing Sertoli cells may be not sufficient to support the massive germ cells in the lumen, which may ultimately cause the germ cell loss and ultimately lead to fewer sperm production in the adult mice. Taken together, these findings suggest that the compromised Sertoli cell proliferation led to the smaller testes and low sperm count in adult cKO mice (Figure S2).

Previous studies have explored the roles of the TGFβ superfamily in embryonic testis cord development. The activation of serine/threonine kinase domain of TGFβ receptors causes phosphorylation of intracellular Smad signal transducer proteins, such as receptor-regulated Smad proteins (SMAD2) and common-Smad (SMAD4) [32]. Of note, when 12.5 dpc testes were treated with TGFβ receptor inhibitors, the testis cords exhibited a stunted and wider appearance, which is similar to those of Sertoli cell specific *Smad4* knockout testes [9,11]. In cells, histone-modifying enzyme SET8 methyltransferase induction is important for inactivation of SMAD2, and efficient degradation of SET8 at the onset of S phase is required for cell cycle progression [28,30]. Additionally, targeted degradation of SET8 during S phase can be achieved by the CRL4 ubiquitin ligase [29]. Here, we demonstrate that SET8 protein level has abruptly accumulated and coincides with the significant decrease of phosphated SMAD2 in the cKO testes, which suggests that as the adapter of CRL4 ubiquitin ligase, DDB1 plays an important role in the Sertoli cell cell-cycle progression by regulating SMAD2 activation and its deficiency ultimately leads to testis cord coiling disruption. Furthermore, it is worth mentioning that the Sertoli proliferation capacity in the 0 dpp *Ddb1* cKO mice is just decreased but not lost (Figure 5), which implies that some TGFβ independent, at least, SMAD2-independent pathways may also participate in the testis cord development. 

## 5. Conclusions

Our experimental findings demonstrated that DDB1 deletion in Sertoli cells compromises Sertoli cell proliferation and disturbs testis cord coiling, and thus causes small testis and decreased sperm production in adult mice. Strikingly, DDB1 dependent canonical TGFβ pathway activation in Sertoli cell is essential for testis cord development. To our knowledge, this is the first upstream regulators of TGFβ pathway in Sertoli cells, and therefore it furthers our understanding of testis cord development. 

## Supplementary Materials

The following are available online at https://www.mdpi.com/2073-4425/10/12/974/s1, Figure S1: High purity of the isolated germ and Sertoli cells, Figure S2: Decreased testis size and weight in prepubertal and adult *Ddb1* cKO mice.
