# Supplementary material for: DDB1 Regulates Sertoli Cell Proliferation and Testis Cord Remodeling by TGFβ Pathway
Source: Genes (Basel). 2019 Nov 26;10(12):974. doi: 10.3390/genes10120974 (PMC6947845; doi:10.3390/genes10120974)
Supplement: Supplementary file 1 [file genes-10-00974-s001.pdf]

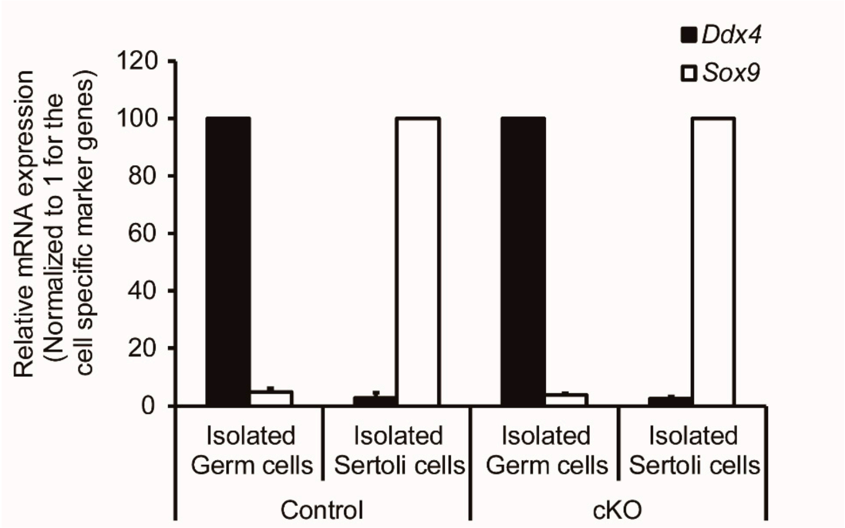

Figure S1. High purity of the isolated germ and Sertoli cells. The purity of isolated germ and Sertoli cells was determined by comparing mRNA levels of cell-specific genes (*Sox9* for Sertoli cells and *Ddx4* for germ cells) using real-time PCR. The mRNA levels were normalized against *Gapdh*, and those for cell specific genes in the isolated cell subsets were arbitrarily set as 1. Data were presented as mean ± S.E.M.

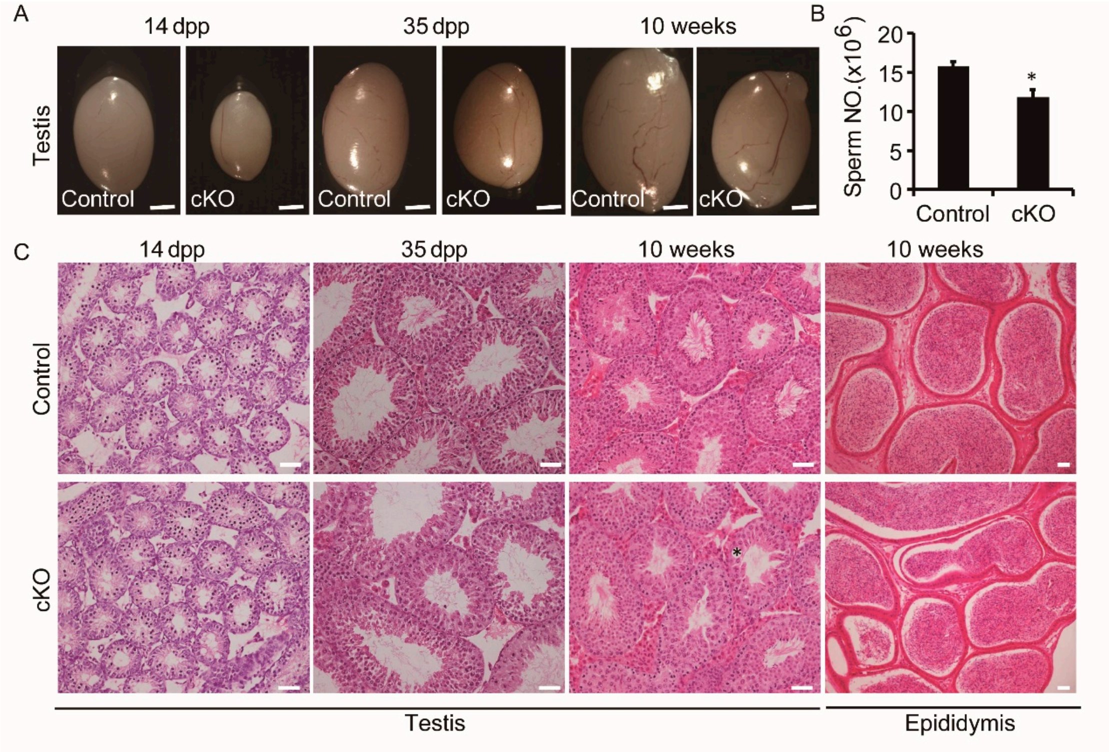

Figure S2. Decreased testis size and weight in prepubertal and adult *Ddb1* cKO mice. (**A**) Representative image of testes from control and cKO mice. Scale bars = 2 mm. (**B**) Sperm number in epididymis from 10 weeks control and cKO mice. Data were presented as mean±S.E.M. *$p < 0.05$, Student's *t*-test; (**C**) H&E staining of the testes from control and cKO mice. Black star indicates vacuolated seminiferous tubules. Scale bars = 50 μm.
